# The Association Between Ventricular Fibrillation and Serum Catecholamine Levels

**DOI:** 10.7759/cureus.43252

**Published:** 2023-08-10

**Authors:** Kiyohiro Oshima, Yusuke Sawada, Yuta Isshiki, Yumi Ichikawa, Kazunori Fukushima, Yuto Aramaki

**Affiliations:** 1 Department of Emergency Medicine, Gunma University Graduate School of Medicine, Maebashi, JPN

**Keywords:** ventricular fibrillation, serum catecholamine level, epinephrine, cardiopulmonary resuscitation, cardiopulmonary arrest

## Abstract

Background and objective

Epinephrine (Ep) is the first choice as a vasoconstrictor in cardiopulmonary resuscitation (CPR) for patients with cardiopulmonary arrest (CPA); however, the Ep concentration in the serum of CPA patients is still unclear. The aim of this study was to evaluate the association between serum Ep levels and achieving the return of spontaneous circulation (ROSC) in out-of-hospital cardiac arrest (OHCA) patients with ventricular fibrillation (VF).

Methods

This was a prospective, observational clinical study involving OHCA patients with VF transferred to our hospital from July 2014 to July 2017. The measurement of serum catecholamines [Ep, norepinephrine (Nep), and dopamine (DOA)] and vasopressin [antidiuretic hormone (ADH)] levels was performed with blood samples obtained immediately upon patients' arrival at our hospital. Patients were classified into two groups: the ROSC(+) group and ROSC(−) group; the serum concentrations of catecholamines and ADH were compared between these two groups.

Results

The serum Ep and Nep levels were lower in the ROSC(+) group than those in the ROSC(−) group and the difference was statistically significant. On the other hand, no significant differences were found in serum DOA and ADH levels between the two groups.

Conclusions

The results of this study suggest that an increment in serum Ep levels does not promote achieving ROSC in OHCA patients with VF.

## Introduction

Epinephrine (Ep) is the drug of choice as a vasoconstrictor in cardiopulmonary resuscitation (CPR) for patients with cardiopulmonary arrest (CPA). As per the Adult Advanced Life Support guidelines published by the 2020 International Consensus on CPR and Emergency Cardiovascular Care (ECC) Science With Treatment Recommendations, the administration of Ep during CPR is strongly recommended based on low-to-moderate certainty of evidence [1,2]. Administration of Ep is recommended as soon as feasible during CPR for patients with non-shockable rhythms, such as pulseless electric activity and asystole. Moreover, administration of Ep is recommended after unsuccessful initial defibrillation attempts during CPR for patients with shockable rhythms, such as ventricular fibrillation (VF) and pulseless ventricular tachycardia. In other words, the significance of Ep as a vasoconstrictor in CPR varies depending on the electrocardiographic waves. Hence, defibrillation is prioritized over the administration of Ep in CPR for patients with shockable rhythms.

The evaluation of Ep concentration in the serum is useful for recognizing the importance of Ep administration in cardiac arrest patients with shockable rhythms. Previously, we evaluated Ep levels in the serum of out-of-hospital cardiac arrest (OHCA) patients with cardiogenic causes. We reported that increased serum Ep levels were not associated with achieving the return of spontaneous circulation (ROSC) [3]. However, the Ep concentration in the serum of OHCA patients with shockable rhythms still remains unclear. In light of this, the aim of this study was to evaluate the relationship between serum Ep levels and achieving ROSC in OHCA patients with VF.

## Materials and methods

This was a prospective, observational clinical study and was approved by the Ethics Committee of Gunma University Hospital (approval no: 14-13). The relatives of patients with CPA provided written informed consent.

**Figure 1:**
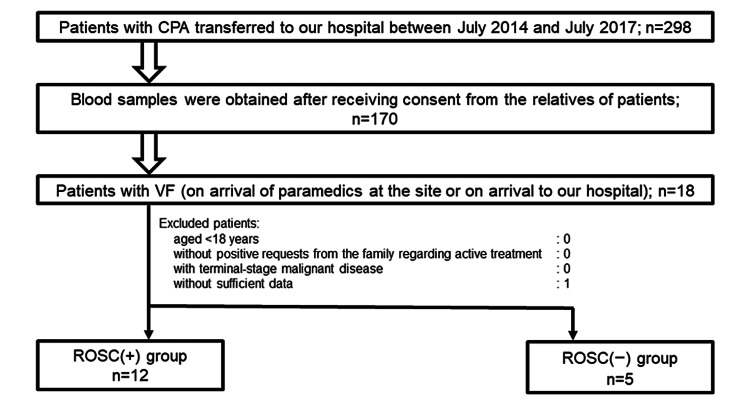
Study flowchart CPA: cardiopulmonary arrest; ROSC: return of spontaneous circulation; VF: ventricular fibrillation

Among the OHCA patients transferred to our hospital from July 2014 to July 2017, those with VF (on the arrival of paramedics at the site or on patients' arrival to our hospital) were included in this study. Figure 1 shows the study flowchart, including the exclusion criteria. We took patients’ blood samples immediately after their arrival at our hospital. Those samples were centrifuged at 3,000 rpm for five minutes, and the serum samples were preserved at −80 °C. The levels of catecholamines such as Ep, norepinephrine (Nep), and dopamine (DOA) in the serum were measured using high-performance liquid chromatography and radioimmunoassay (Bio Medical Laboratories Inc., Tokyo, Japan). The levels of vasopressin [antidiuretic hormone (ADH)] in the serum were also measured with the same method.

Patients were classified into two groups: the ROSC(+) group and ROSC(−) group. In this study, ROSC(+) was defined as the detection of a pulse at the carotid, femoral, or radial artery under advanced CPR, and subsequent maintenance of systolic pressure ≥80 mmHg for at least one hour with or without continuous intravenous or intraosseous administration of vasoconstrictive agents [3-5]. Patients who did not achieve the above condition were considered ROSC(−).

The levels of catecholamines and ADH in the serum, as well as the conditions of resuscitation before and after arrival at our hospital, were compared between the two groups. We determined the causes of cardiac arrest based on the conditions at the time of first contact with the patient, comorbidities (if known), and the results of blood examinations and imaging examinations such as ultrasonography and CT. CT imaging was performed for all patients included in this study, and the images were evaluated by multiple radiologists in our hospital.

Statistical analysis

Qualitative parameters were expressed as counts, numbers, and percentages. On the other hand, quantitative parameters were shown as medians and interquartile ranges (IQR). Comparisons of continuous variables between the two groups were performed using the Mann-Whitney U test. Categorical variables between the two groups were compared using the chi-squared test or Fisher's exact test. We used IBM SPSS Statistics version 25.0 software (IBM Japan, Tokyo, Japan) for statistical analysis, with a p-value <0.05 indicating statistical significance.

## Results

Seventeen patients with VF were evaluated in this study, as shown in Figure 1. A comparison of the background data of the patients is shown in Table 1.

**Table 1:** Comparison of patient backgrounds between the two groups CPR: cardiopulmonary resuscitation; EMS: emergency medical services; Ep: epinephrine; IQR: interquartile range

Variables	ROSC(+) (n=12)	ROSC(−) (n=5)	P-value
Age, years, median (IQR)	66.0 (59.3, 68.3)	73.0 (66.0, 77.0)	0.234
Male/female ratio	11/1	4/1	0.496
The implementation rate of bystander CPR, % (n)	58.3% (7/12)	60.0% (3/5)	0.949
Prehospital intervention of Dr. Heli or Dr. Car, % (n)	16.7% (2/12)	60.0% (3/5)	0.074
The duration between the emergency call and the EMS arrival at the scene, minutes, median (IQR)	6.0 (4.5, 7.0)	8.0 (6.0, 9.5)	0.280
Administered dosage of Ep before obtaining blood samples, mg, median (IQR)	0 (0, 1.0)	4.0 (1.0, 5.0)	0.048
Prehospital resuscitation time, minutes, median (IQR)	13 (8, 23)	25 (19, 48)	0.195
Total administered dosage of Ep, mg, median (IQR)	0 (0, 0.13)	7.0 (6.0, 7.0)	0.006
Total resuscitation time, minutes, median (IQR)	13 (8, 30)	51 (46, 62)	0.006
The causes of cardiac arrest, n			0.031
Cardiogenic	11	2	
Acute aortic dissection	0	2	
Brain hemorrhage	1	0	
Pneumonia	0	1	

There were no significant differences in terms of age, male/female ratio, the implementation rate of bystander CPR, prehospital intervention of Dr. Heli or Dr. Car, duration from the emergency call to the arrival of emergency medical services at the scene, and prehospital resuscitation time between the two groups. However, the administered dosage of Ep before the collection of blood samples was significantly higher in the ROSC(−) group compared to the ROSC(+) group. Finally, the total resuscitation time was significantly longer and the total administered dosage of Ep was significantly higher in the ROSC(−) group compared to the ROSC(+) group. The final diagnoses regarding the causes of cardiac arrest are also shown in Table 1. There was a significant difference in the causes of cardiac arrest, and the rate of cardiogenic cardiac arrest was higher in the ROSC(+) group than in the ROSC(−) group.

**Figure 2:**
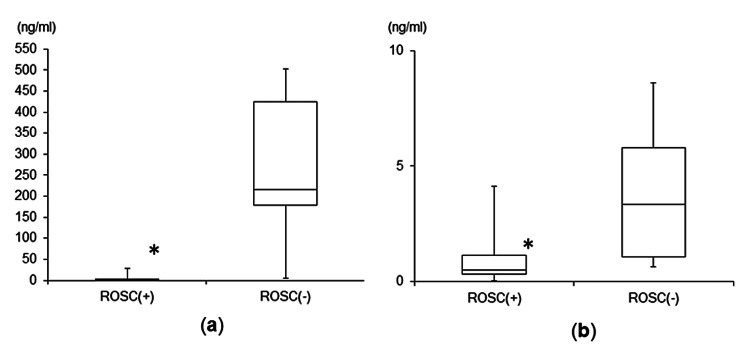
Comparisons of serum Ep and Nep levels between the two groups (a) Comparison of serum Ep levels between the two groups. (b) Comparison of serum Nep levels between the two groups *p<0.05 Ep: epinephrine; Nep: norepinephrine; ROSC: return of spontaneous circulation

The serum Ep levels were significantly lower in the ROSC(+) group versus the ROSC(−) group [normal range of serum Ep concentration: ≤0.10 ng/ml; ROSC(+) group: 0.50 (0.34, 1.93) ng/ml; ROSC(−) group: 216.69 (178.75, 424.93) ng/ml; p=0.002] (Figure 2a). In addition, the serum Nep levels were significantly lower in the ROSC(+) group versus the ROSC(−) group [normal range of serum Nep concentration: 0.10-0.50 ng/ml; ROSC(+) group: 0.50 (0.32, 1.16) ng/ml; ROSC(−) group: 3.34 (1.09, 5.79) ng/ml; p=0.042] (Figure 2b).

**Figure 3:**
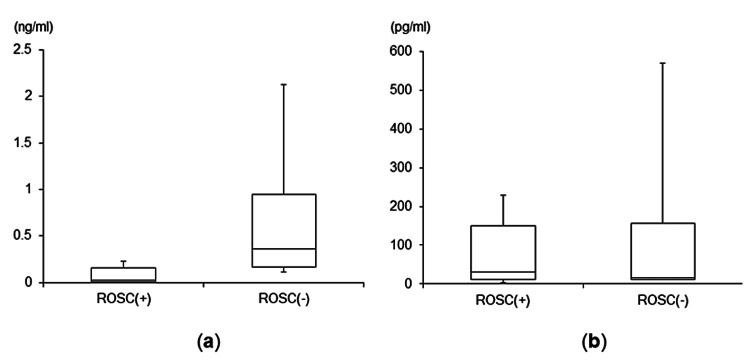
Comparisons of serum DOA and ADH levels between the two groups (a) Comparison of serum DOA levels between the two groups. (b) Comparison of serum ADH levels between the two groups ADH: antidiuretic hormone; DOA: dopamine; ROSC: return of spontaneous circulation

In contrast, there were no significant differences in serum DOA and ADH levels between the two groups [normal range of serum DOA concentration: ≤0.03 ng/ml; ROSC(+) group: 0.02 (0.01, 0.155) ng/ml; ROSC(−) group: 0.37 (0.16, 0.95) ng/ml; p=0.191; normal range of serum ADH concentration: ≤4.2 pg/ml; ROSC(+) group: 30.20 (10.75, 150.55) pg/ml; ROSC(−) group: 15.25 (11.00, 157.13) pg/ml; p=1.000] (Figures 3a, 3b).

## Discussion

The results of this study showed that the serum Ep levels, measured using blood samples obtained immediately after arrival at our hospital, were significantly lower in the ROSC(+) group versus the ROSC(−) group of OHCA patients with VF. The serum Nep levels exhibited a similar tendency. However, there were no significant differences in the serum DOA and ADH levels between the two groups.

In 1963, using animal models and illustrative cases, Pearson and Redding showed the efficacy of Ep in CPR for CPA [6-8]. Otto et al. investigated the mechanism of action of Ep in CPR using a dog model of VF with ischemic myocardium [9,10]. They reported that Ep did not ameliorate the success of countershock during CPR. Nevertheless, it increased the rate of successful ROSC after defibrillation. Recently, Evans et al. studied the administration of Ep before defibrillation for the treatment of in-hospital cardiac arrest due to shockable rhythm using 2000-2018 data from 497 hospitals participating in the American Heart Association’s Get With The Guidelines-Resuscitation registry. The investigators reported that one in five patients was treated with Ep before defibrillation. They concluded that the Ep use before defibrillation was associated with worse survival outcomes [11]. Taking these findings into account, the administration of Ep to patients with VF may be effective only in the setting of defibrillation. In addition, the increase in serum Ep levels caused by the Ep administration may not be related to achieving ROSC in patients with VF. The 2020 International Consensus on CPR and ECC Science With Treatment Recommendations also suggest the administration of Ep after unsuccessful initial defibrillation attempts during CPR for patients with shockable rhythms, such as VF and pulseless ventricular tachycardia. The present findings may support these guidelines.

The dosage of Ep administration before blood sampling was significantly higher in the ROSC(−) group versus the ROSC(+) group. However, there was no significant difference in prehospital resuscitation time between the two groups. This observation can be attributed to two main factors: (1) the emergency medical system in Japan; and (2) the patient backgrounds in this study. Regarding the emergency medical system in Japan, a license and direct permission from medical doctors are required for paramedics to administer Ep to OHCA patients in the prehospital scenario. Therefore, it is not possible for all paramedics to administer Ep to OHCA patients. Notably, no significant difference in the rate of dispatch of Dr. Heli or Dr. Car was found between the two groups. This indicates that medical doctors are able to reach the scene. Thus, it is possible that the management of patients by paramedics at the scene resulted in differences in the administered Ep dosage before blood sampling.

Concerning patient background, this study included patients with VF at the time of arrival of paramedics at the scene or upon arrival at our hospital. More specifically, patients who had VF when the paramedics arrived at the scene and achieved ROSC through CPR performed by paramedics on the way to the hospital were evaluated in this study. This fact may also be responsible for the differences recorded in the administered Ep dosage before blood sampling.

In this study, the serum Nep levels were significantly lower in the ROSC(+) group versus the ROSC(−) group. Nevertheless, no significant difference in serum DOA levels was found between the two groups. DOA is converted to Nep with the action of DOA β-hydroxylase [12]. It is possible that the metabolism of catecholamines, including the activity of enzymes (e.g., DOA β-hydroxylase and dopa decarboxylase), which have a role in converting dopa into DOA, is responsible for this result. However, we did not evaluate the metabolism of catecholamines in the present study.

According to the 2020 International Consensus on CPR and ECC Science With Treatment Recommendations, the administration of ADH instead of Ep and the addition of ADH to Ep during CPR are not recommended [1,2]. There was no significant difference in serum ADH levels between the two groups in this study. This result indicates that the serum levels of ADH may not be associated with achieving ROSC.

This study has a few limitations. This was a single-center study with a small number of patients and included patients with VF at the time the paramedics arrived at the scene or upon arrival at the hospital. In addition, long-term observations were not conducted. Further studies focusing on the serum levels of catecholamines, including Ep, are required to thoroughly analyze the administration of vasopressors during CPR.

## Conclusions

In OHCA patients with VF, the serum Ep level (measured using blood samples obtained immediately after the arrival at the hospital) was significantly lower in the ROSC(+) group versus the ROSC(−) group. Our findings suggest that an increment in the serum Ep levels does not promote achieving ROSC in OHCA patients with VF.
